# Integrating hypertension screening at the time of voluntary HIV testing among adults in South Africa

**DOI:** 10.1371/journal.pone.0210161

**Published:** 2019-02-08

**Authors:** Paul K. Drain, Ting Hong, Anjum Hajat, Meighan Krows, Sabina Govere, Hilary Thulare, Mahomed Yunus S. Moosa, Ingrid Bassett, Connie Celum

**Affiliations:** 1 Department of Global Health, University of Washington, Seattle, United States of America; 2 Department of Medicine, University of Washington, Seattle, United States of America; 3 Department of Epidemiology, University of Washington, Seattle, United States of America; 4 AIDS Healthcare Foundation, Durban, South Africa; 5 Department of Infectious Diseases, University of KwaZulu-Natal, Durban, South Africa; 6 Department of Medicine, Massachusetts General Hospital, Harvard Medical School, Boston, United States of America; Sefako Makgatho Health Sciences University, SOUTH AFRICA

## Abstract

**Background:**

Guidelines recommend integrating hypertension screening for HIV-infected adults, but blood pressure measurements may be dynamic around the time of HIV testing.

**Methods:**

We measured a seated resting blood pressure in adults (≥18 years) prior to HIV testing, and again after receiving HIV test results, in an ambulatory HIV clinic in KwaZulu-Natal, South Africa. We assessed sociodemographics, smoking, body mass index, diabetes, substance abuse, and anxiety/depression. We used blood pressure categories defined by the Seventh Joint National Committee (JNC 7) classifications, which includes normal, pre-hypertension, stage 1 hypertension, and stage 2 hypertension.

**Results:**

Among 5,428 adults, mean age was 31 years, 51% were male, and 35% tested HIV-positive. Before HIV testing, 47% (2,634) had a normal blood pressure, 40% (2,225) had prehypertension, and 10% (569) had stage 1 or 2 hypertension. HIV-infected adults had significantly lower blood pressure measurements and less hypertension, as compared to HIV-negative adults before HIV testing; while also having significantly elevated blood pressures after HIV testing. In a multivariable model, HIV-infected adults had a 30% lower odds of hypertension, compared to HIV-uninfected adults (aOR = 0.70, 95% CI: 0.57–0.85). In a separate multivariable model, HIV-infected adults with CD4 ≤200 cells/mm^3^ had a 44% lower odds of hypertension (aOR = 0.56, 95% CI: 0.38–0.83), as compared to adults with CD4 >200 cells/mm^3^. The mean arterial blood pressure was 6.5 mmHg higher among HIV-infected adults after HIV testing (p <0.001).

**Conclusions:**

HIV-infected adults experienced a transient blood pressure increase after receiving HIV results. Blood pressure measurements may be more accurate before HIV testing and repeated blood pressure measurements are recommended after ART initiation before formally diagnosing hypertension in HIV-infected adults.

## Introduction

Cardiovascular disease (CVD) is a leading cause of mortality worldwide, and a leading cause of HIV/AIDS-related mortality [1,2]. As more HIV-infected adults benefit from expanded access to antiretroviral therapy (ART), more people may experience morbidity and mortality from CVD and chronic non-communicable diseases (NCDs) [3,4]. The World Health Organization (WHO) and others recommend blood pressure screening at HIV diagnosis in order to integrate management of HIV and chronic NCDs [5–7]. By 2025, an estimated three-quarters of people with hypertension will be living in low- and middle-income countries where HIV is currently endemic [8,9]. Globally, an estimated 22% of adults age 18 years or older have hypertension, while the prevalence of hypertension has been estimated to be 34% among South African adults [10,11]. While hypertension management should be integrated with HIV care, busy HIV clinics often measure blood pressure after HIV testing and face difficulties with hypertension management [12].

Similar to the general population, HIV-infected adults should be routinely screened for hypertension, as treatment could be integrated within HIV care [13,14]. In a recent meta-analysis, blood pressure was lower among HIV-infected adults receiving ART, as compared to HIV-uninfected adults [15]. However, these differences may be due to ART medications that have been associated with changes in blood pressure [16,17]. Screening for chronic NCDs at the time of HIV testing may be useful for identifying hypertension among both HIV-infected patients before ART initiation, as well as among HIV-uninfected adults. Since both hypertension and HIV infection are risk factors for CVD [18,19], guidance is needed for how to best integrate hypertension screening and treatment within HIV testing and care [5]. Hypotension is associated with advanced HIV infection, and a study of ART-naïve HIV-infected patients in Tanzania suggested that lower CD4 count and more advanced disease were associated with lower rates of hypertension [20]. While there are biological reasons that blood pressure may be lower in HIV-infected adults with more advanced disease, a person’s blood pressure might be transiently increased after learning of their HIV+ test results.

Currently, there is limited information and guidance on how HIV-infection and associated immunosuppression influences hypertension screening result, and whether blood pressure measurements after receiving an HIV diagnosis causes transient blood pressure changes. Therefore, we sought to understand the association between HIV infection, hypertension, and blood pressure variability among South African adults accessing HIV testing services in a poor urban township. Since hypertension screening routinely occurs after HIV testing, the goal of this study was to determine the degree to which blood pressure measurements and hypertension screening are dynamic around the time of HIV testing in South Africa.

## Methods

### Study design and participants

We conducted a study among adults presenting for voluntary HIV counseling and testing in the outpatient department of the iThembalabantu People’s Hope Clinic (IPHC) in the Umlazi township of South Africa from September 2013 to April 2017. The IPHC tests adults for HIV and provides free clinic- and community-based HIV care and treatment for over 10,000 HIV-infected patients. We enrolled English or Zulu speaking adults ≥18 years of age, who presented to the clinic for HIV testing and were willing and able to provide written informed consent for study participation. We excluded patients known to be pregnant. All participants provided written, informed consent in either English or Zulu. The study was approved by the institutional review board of the University of Washington in Seattle (IRB #49563) and the Medical Research Ethics Committee of the University of KwaZulu-Natal in Durban (Protocol #BF052/13).

### Data collection

The study team enrolled eligible participants prior to HIV testing and completed a questionnaire with demographic, socioeconomic, and mental health questions. All participants were asked about any recent (within 1 month) or prior (>1 month) use for smoking, alcohol use, intravenous drug use, and cannabis use. Within the baseline questionnaire, before HIV testing, we assessed general anxiety using the standardized generalized anxiety disorder 7-item (GAD-7) scale [21], and depression using the patient health questionnaire-9 (PHQ-9) scale [22]. We measured food insecurity using the Household Food Insecurity Access Scale (HFIAS) survey [23], which consists of nine questions on a 3-point Likert scale about perceptions and behavior responses about their food vulnerability. After the interview and following at least 10 minutes of rest, a research assistant obtained a resting, seated blood pressure measurement with feet and back supported using a standardized digital blood pressure machine (7 series wrist blood pressure monitor; Omron Healthcare Inc., Kyoto, Japan).

After blood pressure measurement, all participants were tested for HIV infection using a rapid fingerprick test by an HIV counselor. All positive tests were confirmed with a second rapid test, and participants received pre- and post-test counseling. Only HIV-infected participants were subjected to additional clinical evaluations, including a repeat blood pressure measurement, and blood tests. Participants who tested HIV-negative were advised to return in several months for repeat HIV screening.

Among HIV-infected participants, a research nurse administered a clinical questionnaire and obtained anthropometric measurements using standard procedures. Body weight was measured to the nearest 0.5 kg with the participant in light clothing and using a standardized scale (Seca Inc., Chino, USA). Height was measured to the nearest 0.5 cm using a standiometer with participants wearing no shoes. At the end of the clinical visit and after at least 10 minutes of rest, another seated blood pressure measurement was obtained. All HIV-infected participants had a CD4+ T-cell count, which was performed at the National Health Laboratory Service lab at Prince Myshenyi Hospital using FACS Calibur System (BD, San Jose, CA). All HIV-infected participants received routine HIV medical care, including CD4 count testing and initiation of ART, according to current South African guidelines [24].

### Primary outcome and covariate definitions

The primary outcome in this analysis was blood pressure, and we used the hypertension categories from the Seventh Joint National Committee (JNC 7) [25], which were supported by South African, WHO and CDC hypertension guidelines [26–28]. Normal blood pressure was defined as systolic (SBP)/diastolic (DBP) <120/80 mmHg; prehypertension was defined as SBP 120–139 mmHg or DBP 80–89 mmHg; stage 1 hypertension was defined as SBP 140–159 mmHg or DBP 90–99 mmHg; and stage 2 hypertension was defined as SBP ≥160 mmHg or DBP ≥100 mmHg. We use “hypertension” to refer to a participant having either stage 1 or stage 2 hypertension. We calculated mean arterial blood pressure (MAP) as [(2*diastolic) + systolic]/3].

Body mass index (BMI) was calculated as weight in kilograms divided by the square of height in meters (kg/m^2^), and we used the following standardized definitions: overweight was BMI 25.0–29.9 kg/m^2^ and obesity was BMI ≥30 kg/m^2^ [29]. General anxiety was defined as a GAD-7 score ≥10 [21]. Depression was defined as a PHQ-9 score ≥15, which indicates either “moderately severe” or “severe” depression [22]. Food insecurity was measured using a standardized instrument and scored as food secure, or mild, moderate, or severe food insecurity [23].

### Statistical analyses

We described demographic, socioeconomic, and cardiometabolic characteristics using mean or percentage, and used chi-square tests, Fisher’s exact tests and paired t-tests where appropriate. For changes in blood pressure before and after HIV testing, we used paired t-tests. We used the one-way ANOVA test to compare mean blood pressures across CD4 count categories. Wald tests for association were used in our univariate and multivariable analyses. Logistic regression was used to test associations between hypertension and potential correlates in univariate and multivariable models. Results were reported as odds ratios (ORs) with 95% confidence intervals (CIs). Because receiving positive HIV status results might artificially elevate patient blood pressure readings, blood pressure measurements taken before HIV testing were used for all analyses. In the multivariable models, variables were selected using a backwards selection approach, and any primary exposure of interest was added after the model was completed. All multivariable analyses were adjusted by age and sex. We calculated 95% confidence intervals (CI), reported two-tailed p-values (α = 0.05), and used SAS 9.4 (Cary, USA).

### Role of the funding source

The funder of the study (US National Institutes of Health) had no role in study design, data collection, data analysis, data interpretation, or writing of the report.

## Results

We enrolled 5,618 participants, and excluded 190 participants from the analysis who did not have complete blood pressure measurements (S1 Data). Among the remaining 5,428 participants, mean age was 31.0 years (SD ±10), and 2,681 (49.4%) were female (Table 1). Most participants were never married (91.9%), and over half had completed high school or higher (54.9%). The majority of participants were not currently employed and had an income <2,000 ZAR/month (~US $150/month). Among mental health measures, 26.6% of participants had anxiety and 6.2% had moderate or severe depression. Cigarettes and alcohol were the most commonly used substances within the prior month (22.4% and 34.6% respectively), with any history of injection drug use being rare (1.0%). Overall, 1,905 participants (35.1%) screened tested positive for HIV, among whom the median CD4 count was 307 cells/mm^3^ (interquartile range: 162–481 cells/mm^3^).

**Table 1:** Characteristics of the adult study population presenting for HIV screening in Durban, 2013–2017 (N = 5,428).

	N (%)
***Demographics***	
**Age (years)**	
18–24	1,654 (30.5%)
25–34	2,347 (43.2%)
35–44	882 (16.2%)
≥45	545 (10.0%)
**Sex**	
Female	2,681 (49.4%)
Male	2,747 (50.6%)
***Socioeconomics***	
**Marital status**	
Married	402 (7.4%)
Never married	4,991 (91.9%)
Widowed/divorced	35 (0.6%)
**Number of children**	
No children	1,683 (31.0%)
1 child	1,665 (30.7%)
>1 child	2,060 (38.0%)
**Education**	
None	1,072 (19.7%)
Primary school or some high school	1,375 (25.3%)
High school completion or higher	2,981 (54.9%)
**Current employment**	
Unemployed	3,252 (59.9%)
<20 hours/week	1,872 (34.5%)
≥20 hours/week	304 (5.6%)
**Income**	
<2,000 ZAR/month (~150 USD/month)	4,427 (81.6%)
2,000–10,000 ZAR/month	923 (17.0%)
≥10,000 ZAR/month (~750 USD/month)	51 (0.9%)
**Food insecurity**	
Food secure	4,895 (90.2%)
Food insecure (mild, moderate, severe)	533 (9.8%)
**Transportation method**	
Walk	4,000 (73.7%)
Public transport, private car, other	1,411 (26.0%)
***Mental Health***	
**Anxiety**	
No (GAD-7 score <10)	3,984 (73.4%)
Yes (GAD-7 score ≥10)	1,444 (26.6%)
**Depression**	
No (PHQ-9 score <15)	5,092 (93.8%)
Yes (PHQ-9 score ≥15)	336 (6.2%)
***Substance Abuse***	
**Cigarette use**	
Never used	4,071 (75.0%)
Some use, but not within last month	135 (2.5%)
Used within last month	1,216 (22.4%)
**Alcohol use**	
Never used	3,251 (59.9%)
Some use, but not within last month	288 (5.3%)
Used within last month	1,878 (34.6%)
**Intravenous drug use**	
Never used	5,367 (98.9%)
Ever used	53 (1.0%)
**Cannabis use**	
Never used	5,063 (93.3%)
Ever used	349 (6.4%)
***Clinical Indicators***	
**HIV status**	
Negative	3,523 (64.9%)
Positive	1,905 (35.1%)

### Blood pressure and prevalence of hypertension

The mean SBP was 118 ±15 mmHg, mean DBP was 72 ±16 mmHg, and mean MAP was 87 ±14 mmHg (Table 2). The mean SBP, DBP, and MAP were all significantly lower among HIV-infected adults, compared to HIV-uninfected adults (p values <0.0001). Among the cohort, 2,634 (46.9%) participants had a normal blood pressure, 2,225 (39.6%) had prehypertension, and 569 (10.2%) had either stage 1 or 2 hypertension. Among HIV-infected adults, 185 of 1,905 (9.4%) participants had hypertension, which was less than HIV-uninfected adults (10.5%; p-value 0.004). Among HIV-infected adults, mean SBP was significantly different when stratified by category of CD4 cell count, with the lowest SBP observed among adults with a CD4 ≤200 cells/mm^3^. However, 7.4% of HIV-infected adults with CD4 ≤200 cells/mm^3^ had stage 1 or 2 hypertension. Among HIV-infected adults with CD4 >200 cells/mm^3^, 10.7% had either stage 1 or 2 hypertension.

**Table 2:** Mean blood pressure and prevalence of hypertension by HIV status.

	Total (n = 5,428)	HIV- (n = 3,523)	HIV+ (n = 1,905)	p-value	HIV+ CD4 ≤200 (n = 500)	HIV+ CD4 >200 (n = 1,102)	p-value
	Mean ±SD or N (%)	Mean ±SD or N (%)	Mean ±SD or N (%)		Mean ±SD or N (%)		
***Blood Pressure (mmHg)***							
Systolic blood pressure	118 ±15	118 ±15	116 ±16	**<0.0001**	115 ± 17	117 ± 15	**0.0076**
Diastolic blood pressure	72 ±16	73 ±15	71 ±18	**<0.0001**	70 ± 18	71 ± 18	0.2396
Mean arterial blood pressure	87 ±14	88 ±13	86 ±14	**<0.0001**	85 ± 15	86 ± 14	**0.0499**
***Hypertension***							
Normal blood pressure	2634 (46.9)	1650 (45.3)	984 (49.7)	**0.0044**	258 (51.6)	548 (49.7)	0.0818
Prehypertension	2225 (39.6)	1489 (40.9)	736 (37.2)		205 (41)	436 (39.6)	
Stage 1 Hypertension	419 (7.5)	289 (7.9)	130 (6.6)		23 (4.6)	90 (8.2)	
Stage 2 Hypertension	150 (2.7)	95 (2.6)	55 (2.8)		14 (2.8)	28 (2.5)	

SD = standard deviation

### Blood pressure screening before and after HIV testing

Among HIV infected participants, blood pressure measurements were significantly higher after participants received their positive HIV test results (Table 3). On average, SBP increased by 2.0 mmHg, DBP increased by 8.8 mmHg, and MAP increased by 6.5 mmHg after HIV testing (p values <0.0001). Among HIV-positive participants, people with a normal blood pressure before HIV testing had a large and significant increase in systolic (+7.1 mmHg), diastolic (+11.3 mmHg), and MAP (+9.9 mmHg) after HIV testing (p-values <0.0001). Participants with prehypertension had a 4.3 mmHg increase in MAP. Among adults with a normal blood pressure before HIV testing, 104 (10.6%) had a blood pressure consistent with stage 1 or stage 2 after HIV testing. Similarly, 209 (28.4%) adults changed from a blood pressure consistent with prehypertension before HIV testing to either stage 1 or stage 2 after HIV testing.

**Table 3:** Change in blood pressure following an HIV screening test among HIV-positive adults (N = 1,898).

Blood pressure category before HIV testing	Before HIV testing (Mean ± SD)	After HIV testing (Mean ± SD)	Mean difference	p-value
**All Participants (n = 1898)**				
Systolic BP (mmHg)	116 ± 16	118 ± 19	1.97	**<0.001**
Diastolic BP (mmHg)	71 ± 18	80 ± 14	8.82	**< .0001**
Mean arterial BP (mmHg)	86 ± 14	92 ± 15	6.52	**< .0001**
**Participants with Normal BP (n = 981)**				
Systolic BP (mmHg)	105 ± 9	113 ± 16	7.13	**< .0001**
Diastolic BP (mmHg)	64 ± 14	76 ± 12	11.30	**< .0001**
Mean arterial BP (mmHg)	78 ± 10	88 ± 12	9.91	**< .0001**
**Participants with Prehypertension (n = 735)**				
Systolic BP (mmHg)	124 ± 7	121 ± 18	-2.75	**< .0001**
Diastolic BP (mmHg)	74 ±17	82 ± 14	7.87	**< .0001**
Mean arterial BP (mmHg)	91 ± 11	95 ± 14	4.33	**< .0001**
**Participants with Stage 1 Hypertension (n = 128)**				
Systolic BP (mmHg)	135 ± 12	132 ± 21	-3.38	**0.056**
Diastolic BP (mmHg)	87 ± 16	90 ± 16	2.90	**0.127**
Mean arterial BP (mmHg)	103 ± 11	104 ± 17	0.81	**0.596**
**Participants with Stage 2 Hypertension (n = 54)**				
Systolic BP (mmHg)	156 ± 22	141 ± 25	-14.87	**0.001**
Diastolic BP (mmHg)	107 ± 15	97 ± 19	-10.15	**0.002**
Mean arterial BP (mmHg)	123 ± 13	120 ± 64	-11.72	**0.0002**

SD = standard deviation

### Risk factors for hypertension

We compared baseline characteristics between normotensive and hypertensive participants among the entire cohort and separately for HIV-infected adults. Among the entire cohort, several characteristics, including older age, male sex, being married, and having more children, were all significantly associated with hypertension. In addition, having anxiety, more alcohol use, and more cannabis use were also associated with more hypertension. Among HIV-infected adults, older age, being married, and having more children were similarly associated with hypertension. In addition, we observed a higher prevalence of hypertension among obese participants and adults with CD4 above 200 cells/mm^3^.

In univariate analyses among the entire cohort, the risk factors for hypertension were older age, male sex, being married, having >1 child, having current employment, having a higher income, having more anxiety, more alcohol use, and more cannabis use (Table 4). In a multivariable model, significant risk factors included older age, anxiety, alcohol use, and HIV status. When controlling for other significant variables, HIV-infected adults had a 30% lower odds of hypertension, as compared to HIV-uninfected adults (aOR = 0.70, 95% CI: 0.57–0.85).

**Table 4:** Unadjusted and adjusted odds ratios (95% confidence intervals) for the association between baseline characteristics and hypertension for the entire study population.

	# hypertensive total at risk	Unadjusted OR (95% CI)	p-value	Adjusted OR (95% CI)	p-value
***Demographics***					
**Age (years)**			**< .0001**		**< .0001**
18–24	96/1,654 (5.8%)	-		-	-
25–34	226/2,347 (9.6%)	1.73 (1.35–2.21)		1.83 (1.42, 2.35)	
35–44	124/882 (14.1%)	2.65 (2.01–3.51)		2.88 (2.16, 3.84)	
≥45	123/545 (22.6%)	4.73 (3.55–6.31)		5.14 (3.83, 6.89)	
**Sex**			**0.0004**		-
Female	241/2,681 (9.0%)	-		-	
Male	328/2,747 (11.9%)	1.37 (1.15–1.64)		-	
***Socioeconomics***					
**Marital status**			**< .0001**		-
Never married	486/4,991 (9.7%)	-		-	
Married	74/402 (18.4%)	2.09 (1.60–2.74)		-	
Widowed/divorced	9/35 (25.7%)	3.21 (1.50–6.89)		-	
**Number of children**			**< .0001**		-
No children	144/1,683 (8.6%)	-		-	
1 child	140/1,665 (8.4%)	0.98 (0.77–1.25)		-	
>1 child	284/2,060 (13.8%)	1.71 (1.38–2.11)		-	
**Education**			0.189		-
None	122/1,072 (11.4%)	-		-	
Primary school or some high school	155/1,375 (11.3%)	0.99 (0.77–1.27)		-	
High school completion or higher	292/2,981 (9.8%)	0.85 (0.68–1.06)		-	
**Current employment**			**0.005**		-
No	319/3,252 (9.8%)	-		-	
Yes, <20 hours/week	202/1,872 (10.8%)	1.11 (0.92–1.34)		-	
Yes, ≥20 hours /week	48/304 (15.8%)	1.72 (1.24–2.40)		-	
**Income**			**0.0008**		-
<2,000 ZAR/month (~150 USD/month)	434/4,427 (9.8%)	-		-	
2,000–10,000 ZAR/month	119/923 (12.9%)	1.36 (1.10–1.69)		-	
≥10,000 ZAR/month (~750 USD/month)	11/51 (21.6%)	2.53 (1.29–4.97)		-	
**Food insecurity**			0.539		-
Food secure	509/4,895 (10.4%)	-		-	
Food insecure (mild, moderate, severe)	60/533 (11.3%)	1.09 (0.82–1.45)		-	
**Transportation method**			0.603		-
Walk	414/4,000 (10.4%)	-		-	
Public transport, private car, other	153/1,411 (10.8%)	1.05 (0.87–1.28)		-	
***Mental Health***					
**Anxiety**			**< .0001**		**0.0001**
No (GAD-7 score <10)	376/3,984 (9.4%)	-		-	-
Yes (GAD-7 score ≥10)	193/1,444 (13.4%)	1.48 (1.23–1.78)		1.47 (1.21, 1.79)	
**Depression**			0.968		-
No (PHQ-9 score <15)	534/5,092 (10.5%)	-		-	
Yes (PHQ-9 score ≥15)	35/336 (10.4%)	0.99 (0.69–1.42)		-	
***Substance Abuse***					
**Cigarette use**			0.117		-
Never used	407/4,071 (10.0%)	-		-	
Some use, but not within last month	18/135 (13.3%)	1.39 (0.83–2.30)		-	
Used within last month	143/1,216 (11.8%)	1.20 (0.98–1.47)		-	
**Alcohol use**			**0.007**		**0.0066**
Never used	310/3,251 (9.5%)	-		-	
Some use, but not within last month	42/288 (14.6%)	1.62 (1.14–2.29)		1.55 (1.08, 2.21)	
Used within last month	215/1,878 (11.4%)	1.23 (1.02–1.47)		1.28 (1.06, 1.54)	
**Intravenous drug use**			0.274		-
Never used	560/5,367 (10.4%)	-		-	
Ever used	8/53 (15.1%)	1.53 (0.72–3.25)		-	
**Cannabis use**			**0.039**		-
Never used	519/5,063 (10.3%)	-		-	
Ever used	48/349 (13.8%)	1.40 (1.02–1.92)		-	
***Clinical Indicators***					
**HIV status**			0.173		**0.0003**
Negative	384/3,523 (10.9%)	-		-	
Positive	185/1,905 (9.7%)	0.88 (0.73–1.06)		0.70 (0.57, 0.85)	

GAD-7 = Generalized Anxiety Disorder 7-item scale; OR = odds ratio; PHQ-9 = Patient Health Questionnaire-9; USD = United States Dollar; ZAR = South African Rand.

Among HIV-infected adult, risk factors for hypertension in univariate analyses included older age, being married, having >1 child, having current employment, having anxiety, more cigarette smoking, more cannabis use, a high BMI, and higher CD4 count (Table 5). A CD4 count ≤200 cells/mm^3^ was associated with significantly lower rates of hypertension, as compared to participants with a CD4 >200 cells/mm^3^. Multivariable analyses among HIV-positive adults suggest that age, anxiety, and CD4 count are independently associated with hypertension. After adjusting for age and anxiety, CD4 count ≤200 cells/mm^3^ was found to be associated with 44% lower odds of hypertension compared to CD4 >200 cells/mm^3^ (aOR = 0.56, 95% CI: 0.38, 0.83, p = 0.004). In sub-analyses, participants who had no prior HIV testing did not have a higher prevalence of hypertension, as compared to participants who previously tested HIV-positive.

**Table 5:** Unadjusted and adjusted odds ratios (95% confidence intervals) for the association between baseline characteristics and hypertension for HIV+ participants.

	# hypertensive total at risk	Unadjusted OR (95% CI)	p-value	Adjusted OR (95% CI)	p-value
***Demographics***					
**Age (years)**			**< .0001**		**< .0001**
18–24	21/346 (6.1%)	-		-	
25–34	72/901 (8.0%)	1.34 (0.81–2.22)		1.45 (0.80–2.60)	
35–44	56/448 (12.5%)	2.21 (1.31–3.73)		2.70 (1.48–4.94)	
≥45	36/210 (17.1%)	3.20 (1.81–5.66)		3.91 (2.03–7.52)	
**Sex**			**0.063**		-
Female	99/1,141 (8.7%)	-		-	
Male	86/764 (11.3%)	1.34 (0.98–1.81)		-	
***Socioeconomics***					
**Marital status**			**0.014**		-
Never married	162/1,761 (9.2%)	-		-	
Married	19/129 (14.7%)	1.70 (1.02–2.85)		-	
Widowed/divorced	4/15 (26.7%)	3.59 (1.13–11.41)		-	
**Number of children**			**0.001**		-
No children	20/336 (6.0%)	-		-	
1 child	51/632 (8.1%)	1.39 (0.81–2.37)		-	
>1 child	113/930 (12.2%)	2.19 (1.33–3.58)		-	
**Education**			0.736		-
None	44/438 (10.0%)	-		-	
Primary school or some high school	59/657 (9.0%)	0.88 (0.59–1.33)		-	
High school completion or higher	82/810 (10.1%)	1.01 (0.69–1.48)		-	
**Current employment**			**0.036**		-
No	102/1,092 (9.3%)	-		-	
Yes, <20 hours/week	62/684 (9.1%)	0.97 (0.69–1.35)		-	
Yes, ≥20 hours/week	21/129 (16.3%)	1.89 (1.13–3.14)		-	
**Income**			0.095		-
<2,000 ZAR/month (~150 USD/month)	137/1,520 (9.0%)	-		-	
2,000–10,000 ZAR/month	42/363 (11.6%)	1.32 (0.92–1.91)		-	
≥10,000 ZAR/month (~750 USD/month)	3/13 (23.1%)	3.03 (0.82–11.14)		-	
**Food insecurity**			0.431		-
Food secure	161/1,691 (9.5%)	-		-	
Food insecure (mild, moderate, severe)	24/214 (11.2%)	1.20 (0.76–1.89)		-	
**Transportation method**			0.992		-
Walk	132/1,361 (9.7%)	-		-	
Public transport, private car, other	52/537 (9.7%)	1.00 (0.71–1.40)		-	
***Mental Health***					
**Anxiety**			**0.019**		**0.010**
No (GAD-7 score <10)	103/1,212 (8.5%)	-		-	-
Yes (GAD-7 score ≥10)	82/693 (11.8%)	1.45 (1.06–1.96)		1.56 (1.11–2.18)	
**Depression**			0.973		-
No (PHQ-9 score <15)	167/1,721 (9.7%)	-		-	
Yes (PHQ-9 score ≥15)	18/184 (9.8%)	1.01 (0.60–1.68)		-	
***Substance Abuse***					
**Cigarette use**			**0.014**		-
Never used	124/1,439 (8.6%)	-		-	
Some use, but not within last month	4/46 (8.7%)	1.01 (0.36–2.86)		-	
Used within last month	56/417 (13.4%)	1.65 (1.18–2.30)		-	
**Alcohol use**			0.061		-
Never used	98/1,163 (8.4%)	-		-	
Some use, but not within last month	15/118 (12.7%)	1.58 (0.89–2.83)		-	
Used within last month	71/618 (11.5%)	1.41 (1.02–1.95)		-	
**Intravenous drug use**			0.631		-
Never used	182/1,887 (9.6%)	-		-	
Ever used	2/15 (13.3%)	1.44 (0.32–6.44)		-	
**Cannabis use**			**0.002**		-
Never used	164/1,789 (9.2%)	-		-	
Ever used	20/110 (18.2%)	2.20 (1.32–3.67)		-	
***Clinical Indicators***					
**Glucose level**			0.076		-
<140 mg/dl	103/1,120 (9.2%)	-		-	
≥140 mg/dl	6/32 (18.8%)	2.28 (0.92–5.66)		-	
**BMI (kg/m**^**2**^**)**			**0.009**		-
Below 18.5	12/126 (9.5%)	1.22 (0.64–2.32)		-	
18.5–24.9	69/869 (7.9%)	-		-	
25.0–29.9	44/489 (9.0%)	1.15 (0.77–1.70)		-	
30.0 and above	58/417 (13.9%)	1.87 (1.29–2.71)		-	
**CD4 count**			**0.039**		**0.004**
≤ 200	37/500 (7.4%)	-		-	
> 200	118/1102 (10.7%)	1.5 (1.02, 2.21)		1.78 (1.20, 2.64)	

GAD-7 = Generalized Anxiety Disorder 7-item scale; OR = odds ratio; PHQ-9 = Patient Health Questionnaire-9; USD = United States Dollar; ZAR = South African Rand.

## Discussion

In this cross-sectional study of ART-naïve South African adults, 10.2% had either stage 1 or 2 hypertension. HIV infection and immunosuppression (CD4 ≤200 cells/mm^3^) were associated with significantly lower baseline rates of hypertension, when adjusted for other contributing factors. Importantly, HIV-positive adults experienced a significant transient increase in blood pressure after receiving an HIV+ test result. Our results demonstrate that blood pressure measurements are dynamic after HIV testing, suggesting that hypertension screening should ideally occur before HIV testing and repeated measures are necessary after ART initiation before formally diagnosing hypertension in HIV-infected adults. These findings should help inform ongoing efforts to integrate non-communicable disease screening with HIV care and treatment in resource-limited settings.

Several studies and a recent meta-analyses have reported differences in hypertension for HIV-infected adults receiving ART and HIV-negative adults [15,30–34]. A Ugandan study reported increased risk of hypertension after initiation of ART among adults with low nadir CD4 counts [35], and a US-based study found longer ART use was associated with higher incidence of hypertension [36]. Surveys in South Africa have reported conflicting results when comparing hypertension rates between HIV-negative adults and untreated HIV-infected adults [37,38]. One study found adults with CD4 <50 cells/mm^3^ was positively associated with risk of sustained hypertension [39], while several additional studies found no association between CD4 cell count and hypertension [40–42]. No studies, to our knowledge, have reported on hypertension screening both before and after HIV testing among HIV-infected adults in sub-Saharan Africa. Our study adds an important comparison between an ART-untreated cohort of HIV-positive individuals and HIV-negative persons. In addition, we assessed the blood pressure changes from receiving an HIV diagnosis, at the time of HIV testing services, which is currently the recommended visit for hypertension screening.

The biological mechanisms underlying the relation between HIV infection and hypertension remain unclear, though several pathways have been suggested, including direct vascular injury by the HIV virus and lipodystrophy, dyslipidemia, direct mitochondrial DNA damage, and insulin resistance resulting from ART use [18,43,44]. Hypertension in these cohorts may either be masked or affected by a combination of undetermined biological and psychological mechanisms, and it is thus increasingly important to screen these patients regularly for CVD risk factors. A study in San Francisco identified two potential biomarkers of cardiovascular risk among HIV-infected patients, which may have potential use in screening HIV-positive patients that are treatment-naïve in addition to regular blood pressure measurements for identifying those at risk for CVDs [45].

Our study had several strengths and limitations. Major strengths included a large sample size allowing more robust analyses of covariates for hypertension and before/after HIV testing, stratifying results by relative immunosuppression, and adjusting multivariable model for important risk factors, such as anxiety. In addition, the prevalence of hypertension among HIV-infected adults was not impacted by participants who already knew their HIV status and were seeking confirmatory HIV testing. Limitations included obtaining only one blood pressure measurement at each time point, which is not consistent with American Heart Association guidelines since one measure may result in misclassification, and using a wrist-based measurement device [46]. However, we did not perform repeated measurements among those HIV-uninfected, since they might be expected to have a lower blood pressure upon repeated testing. While the cross-sectional design prevents us from making claims of causality in the association between HIV status and hypertension, these results reflect the real clinical practice of hypertension screening at HIV testing in South Africa.

In conclusion, our findings suggest that untreated HIV-infected adults, and particularly immunocompromised adults, have lower baseline rates of hypertension compared to HIV-negative adults, and that blood pressure transiently increases after receiving a positive HIV test result. Since hypertension screening may be dynamic around the time of HIV testing, our findings do not support measuring blood pressure following HIV testing during the same clinical visit. Instead, hypertension screening should ideally occur before HIV testing, be repeated again after ART initiation and viral load suppression, and be continued at regular intervals [47]. As ART delivery increase the life expectancy of people living with HIV, providing appropriate diagnosis and management of hypertension will become increasingly important.

## Supporting information

S1 Data A dataset for the study cohort. (XLSX)
